# Supplementary material for: Content-rich biological network constructed by mining PubMed abstracts
Source: BMC Bioinformatics. 2004 Oct 8;5:147. doi: 10.1186/1471-2105-5-147 (PMC528731; doi:10.1186/1471-2105-5-147)
Supplement: Additional File 2 — The original results of the above study (non-essential files are deleted to keep the file size under the limit set by BMC bioinformatics). [file 1471-2105-5-147-S2.bz2 › chilibotAdditionalFile2/dip10/96ID9324938E403/html/BAD_BCL2L1.html]

 


 **BAD** and **BCL2L1** 
  
Found 251 abstracts in PubMed, retrieved 10.  
 

 What does Google say? 
 PDF only 
| .edu only 

---

**Interactive relationship** (e.g. stimulation, inhibition, etc)

**Inhibitory relationship**- CD44v7 deficiency was characterized by an increase in the percentage of apoptotic cells after stimulation, increased numbers of CD95L and CD152 positive cells, low levels of the anti apoptotic proteins Bcl 2 and Bcl Xl  [ **BCL2L1** ] , and decreased phosphorylation of the pro apoptotic protein  **BAD** .  Ref: 12832452 J Leukoc Biol, 2003
**Stimulatory relationship**- In WtFLT3 transfected 32D WtFLT3 32D cells, FLT3 ligand FL stimulation did not restore the down regulation of Bcl XL  [ **BCL2L1** ]  but maintained the phosphorylation of  **Bad** .  Ref: 12842996 Blood< MedlineTA>Blood, 2003
**Neutral relationship**- We describe the implementation of confocal and two photon excitation fluorescence resonance energy transfer FRET microscopy techniques through which we demonstrate interaction between the proapoptotic protein  **BAD**  and the prosurvival protein Bcl xL  [ **BCL2L1** ]  within TAI following TBI.  Ref: 12880338 J Biomed Opt, 2003

**Non-interactive relationship** (e.g. studied together, co-existance, homology, etc.)

- One proposed pathway by which this has been hypothesized to occur is the Ca 2 mediated activation of calmodulin and subsequent activation of the phosphatase calcineurin with dephosphorylation of a protein known as  **BAD** , leading to a proapoptotic interaction between  **BAD**  and the mitochondrial protein Bcl xL  [ **BCL2L1** ] .  Ref: 12880338 J Biomed Opt, 2003
- While this pathway is an intriguing route for traumatic axonal pathogenesis, neither conventional immunocytochemical histochemical nor ultrastructural approaches have had the capacity to shed insight on whether  **BAD**  and Bcl xL  [ **BCL2L1** ]  interact in TAI in vivo.  Ref: 12880338 J Biomed Opt, 2003
- Furthermore, the dephosphorylation of  **Bad**  using LY294002 and PD98059 was insufficient for apoptosis, and the down regulation of Bcl XL  [ **BCL2L1** ]  using antisense treatment was needed to induce apoptosis.  Ref: 12842996 Blood< MedlineTA>Blood, 2003
- In a murine myeloid cell line, 32D, IL 3 deprivation induced apoptosis following the down regulation of Bcl XL  [ **BCL2L1** ]  and the dephosphorylation of  **Bad** .  Ref: 12842996 Blood< MedlineTA>Blood, 2003
- **Bad**  and Bcl X  [ **BCL2L1** ]  mRNA transcription increased significantly.  Ref: 12830043 J Thorac Cardiovasc Surg, 2003
- FLT3 kinase inhibitor, AG1296, alone not only dephosphorylated  **Bad**  but also down regulated Bcl XL  [ **BCL2L1** ] , leading FLT3 ITD 32D cells into apoptosis.  Ref: 12842996 Blood< MedlineTA>Blood, 2003
- Furthermore, Bcl 2 family members  **Bad** , Bak, and Bcl xS  [ **BCL2L1** ]  protein levels were increased in FHIT transfected clones when compared with Panc 1 cells.  Ref: 12890991 Pancreatology, 2003
- Here, we found that HDB induced apoptotic cell death was accompanied by upregulation of cyclin D3, Bax, and p21 and down regulation of Bcl X  [ **BCL2L1** ]  L, while HDB had no effect on the levels of Bcl 2 and  **Bad**  protein.  Ref: 12822933 J Agric Food Chem< MedlineTA>J Agric Food Chem, 2003
- Remarkably, these events occurred in the absence of any reduction in the expression of the Bcl 2 family members Bcl 2, Mcl 1 and Bcl XL  [ **BCL2L1** ] , or any change in the proapoptotic molecules  **Bad**  or Bax.  Ref: 12842827 Am J Physiol Gastrointest Liver Physiol< MedlineTA>Am J Physiol Gastrointest Liver Physiol, 2003
